# Changes in exercise capacity, muscle strength, and health-related quality of life in esophageal cancer patients undergoing esophagectomy

**DOI:** 10.1186/s13102-016-0060-y

**Published:** 2016-11-03

**Authors:** Takayuki Inoue, Satoru Ito, Masahiko Ando, Motoki Nagaya, Hiromichi Aso, Yota Mizuno, Keiko Hattori, Hiroki Nakajima, Yoshihiro Nishida, Yukiko Niwa, Yasuhiro Kodera, Masahiko Koike, Yoshinori Hasegawa

**Affiliations:** 1 Department of Rehabilitation, Nagoya University School of Medicine, Nagoya, 466-8550 Japan; 2 Department of Respiratory Medicine, Nagoya University School of Medicine, Nagoya, 466-8550 Japan; 3 Center for Advanced Medicine and Clinical Research, Nagoya University School of Medicine, Nagoya, 466-8550 Japan; 4 Gastroenterological Surgery II, Nagoya University School of Medicine, Nagoya, 466-8550 Japan

**Keywords:** Esophagectomy, COPD assessment test, Health-related quality of life, Pulmonary rehabilitation, Six-minute walk test

## Abstract

**Background:**

Surgery for cancer of the thoracic esophagus is a challenging procedure associated with high morbidity and mortality. Perioperative rehabilitation has been introduced to promote early mobilization of the patients and to prevent postoperative pulmonary complications. The purpose of the present study was to characterize the preoperative functional exercise capacity, muscle strength, anxiety, depression, and health-related quality of life (QOL) in patients with esophageal cancer, and to evaluate the impact of radical esophagectomy on these parameters.

**Methods:**

We performed a retrospective review of 34 consecutive patients with newly diagnosed resectable esophageal cancer who underwent esophagectomy followed by postoperative rehabilitation from January to December 2014. Patients were tested for 6-min walk distance (6MWD), knee-extensor muscle strength, hand grip strength, the Hospital Anxiety and Depression Scale (HADS), and the chronic obstructive pulmonary disease (COPD) assessment test (CAT) before and two weeks after the surgery. Before surgery, the pulmonary function test, and components of the MOS 36-item Short-Form Health Survey (SF-36) Questionnaire for general health were assessed.

**Results:**

The mean age was 67.3 ± 8.1 years. The patients were predominantly male (76.4 %), had high rates of smoking history (91.2 %), and squamous cell carcinoma (97.1 %). The predicted value for forced expiratory volume in 1 s was 94.0 ± 15.9 %, and 12 patients (35.3 %) had COPD. The clinical stage was 0-I in 12 patients, II in 4 patients, III in 16 patients, and IV in 2 patients. Thirty-one patients (91.2 %) underwent open surgery. At the baseline, components of the SF-36 scores significantly correlated with CAT and HADS scores, and the physical status was significantly poorer in patients with COPD than those without. Comparisons between the preoperative and postoperative values revealed significant decreases in 6MWD, hand grip strength, isometric knee extensor muscle strength, and a significant increase in CAT scores but not in HADS scores after surgery. In multiple regression analysis, decreases in 6MWD after the surgery significantly correlated with the preoperative physical component summary of SF-36.

**Conclusions:**

Our results indicate that surgery remained detrimental to health outcomes at two weeks. Further research should investigate whether prehabilitation would improve the postoperative outcomes, QOL, and physical fitness.

**Electronic supplementary material:**

The online version of this article (doi:10.1186/s13102-016-0060-y) contains supplementary material, which is available to authorized users.

## Background

Esophagectomy is the standard therapy for patients with localized esophageal cancer, but it is a highly invasive procedure and associated with serious postoperative complications such as pulmonary complications, anastomotic leaks, and sepsis [1]. Major pulmonary complications after esophagectomy have been implicated in prolonged hospital stays and postoperative mortality [2, 3]. The preoperative health status is important because advanced age, preoperative chemoradiotherapy, and comorbidity of chronic obstructive pulmonary disease (COPD) are associated with the increased risk of postoperative complications and mortality [4, 5]. Moreover, it has been reported that a lack of preoperative physical activity and the loss of maximum oxygen uptake are risk factors for pulmonary or cardiopulmonary complications [6–8].

Perioperative rehabilitation has been expected to improve physical fitness, promote early mobilization, and reduce postoperative pulmonary complications in patients with esophageal cancer [9, 10]. In order to manage the rehabilitation program, it is important to adequately assess the status of functional exercise capacity and health-related quality of life (QOL) before surgery. Moreover, esophagectomy itself worsens health-related QOL and physical fitness [11–13]. A prospective study by Reynolds et al. demonstrated that esophageal resection had a negative impact on health-related QOL as assessed by the 30-item European Organization for the Research and Treatment of Cancer (EORTC) QOL Core Questionnaires (QLQ) (EORTC QLQ-C30) [14] and esophageal cancer-specific EORTC QLQ (EORTC QLQ-OES24) [15] 3 months after surgery [12]. Moreover, Teoh et al. reported that esophagectomy was associated with worsened physical functioning and fatigue symptoms as assessed by EORTC QLQ-C30 and QLQ-OES24 up to 6 months after treatment [11]. Tatematsu et al. reported that 6MWD and knee-extensor muscle strength were significantly decreased approximately 3 weeks after esophagectomy [13]. However, the pre- and postoperative physical fitness, health-related QOL, psychological aspects, and their relationships have not been fully evaluated in patients with esophageal cancer who have undergone esophagectomy.

The purpose of the present study was to characterize the preoperative functional exercise capacity, muscle strength, anxiety, depression, and health-related QOL in patients with esophageal cancer, and to evaluate the impact of radical esophagectomy on these parameters 2 weeks after the surgery.

## Methods

### Patients

**Fig. 1:**
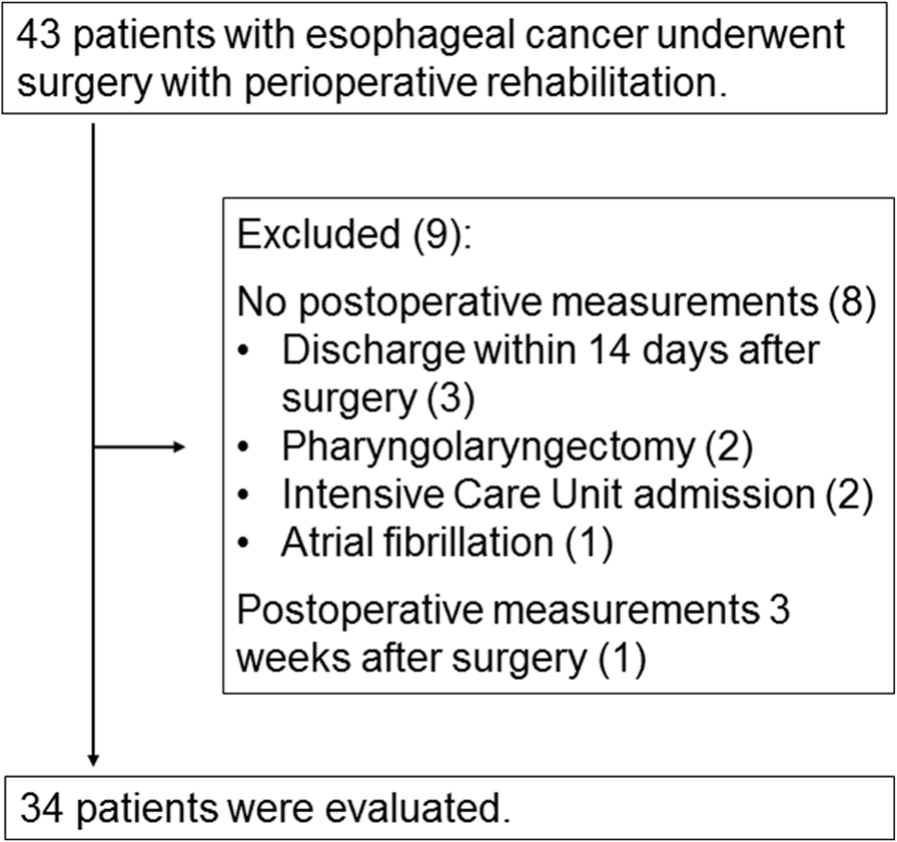
Flow of participants

Records of 43 consecutive patients with esophageal cancer who underwent scheduled radical esophagectomy in the Department of Gastroenterological Surgery II, Nagoya University Hospital from January to December 2014 were retrospectively reviewed. Patients who underwent emergency surgery were not included. At our institution, perioperative pulmonary rehabilitation is routinely performed on patients who underwent esophagectomy. In addition, physical, mental, and QOL status are routinely assessed before and after surgery as the perioperative rehabilitation program according to previous reports and guidelines [2, 10, 13, 16–19]. Patients are allowed to refuse the assessment if they do not wish. A flow chart describing the inclusion process is shown in Fig. 1. Thirty-four patients who underwent pre- and postoperative physical assessment were evaluated. Nine patients were excluded because they were discharged within 14 days after surgery (*n* = 3), were unable to walk without assistance due to invasive pharyngolaryngectomy (*n* = 2), were postoperatively admitted to the Intensive Care Unit (*n* = 2), and had atrial fibrillation (*n* = 1), and physiological status was evaluated 3 weeks after surgery (*n* = 1). The tumors were staged according to the seventh edition of the Union for International Cancer Control TNM staging system, and the tumor grades were classified according to the WHO classification of histological differentiation. The severity of postoperative complications was classified using the Clavien-Dindo classification [20]. Information about patients was collected through a review of electronic medical records.

### Pulmonary function tests

Preoperative pulmonary functions were routinely measured using computerized equipment (Fudak77, Fukuda Sangyo, Tokyo, Japan) at the clinical laboratory within 30 days before the operation. As spirometric parameters, vital capacity (VC), forced vital capacity (FVC), and forced expiratory volume in 1 s (FEV_1_) were measured. COPD was defined as an FEV_1_/FVC ratio <70 % without any other respiratory diseases. The predicted values for spirometry and lung volumes were calculated according to the method of the Japanese Respiratory Society [21].

### Measurements of physical fitness

The 6-min walk distance (6MWD) was measured by the 6-min walk test (6MWT) according to guidelines of the American Thoracic Society [22]. During the 6MWT, oxygen saturation of a peripheral artery (SpO_2_) was measured using a pulse oximeter (Pulsox-Me300; Teijin Pharma Co., Tokyo, Japan) without supplemental oxygen. Desaturation was defined as a fall in SpO_2_ ≥ 4 % or SpO_2_ < 90 % during the 6MWT [23]. Isometric knee extensor muscle strength was assessed using a hand-held dynamometer (Mutus F-100; Anima Co., Tokyo, Japan). Hand grip strength was measured using a digital dynamometer (Grip-D, Takei Co., Niigata, Japan).

### Assessment of anxiety, depression, and health-related QOL

Anxiety and depression were measured using the validated Japanese version of the Hospital Anxiety and Depression Scale (HADS) [24]. The scores range from 0 to 21 for each subscale, with a score ≥8 denoting a probable case. A higher score represents a higher level of anxiety or depression. Health-related QOL was assessed by the validated Japanese version of the MOS 36-item Short-Form Health Survey (SF-36) version 2, which has eight subscales and three component summary scores: a physical component summary (PCS), mental component summary (MCS), and role/social component summary (RCS) [25]. A higher SF-36 score represents a better health-related QOL. Respiratory health-related QOL was assessed by a validated Japanese version of the COPD assessment test (CAT), an eight-item questionnaire including cough, phlegm, chest tightness, breathlessness, activity limitation, confidence to leave home, sleep, and energy [26]. A higher CAT score represents a poorer respiratory health-related QOL.

### Rehabilitation program

Pre- and postoperative rehabilitation was performed by specialized physical therapists (T.I., M.N, Y.M., K.H., and H.N.). The preoperative pulmonary rehabilitation protocol included (1) measurements of 6MWD, knee extensor muscle strength, and hand grip strength, (2) assessment of HADS, SF-36, and CAT, and (3) orientation to the postoperative rehabilitation program and encouragement of early mobilization. Preoperative measurements were assessed 1 to 10 days before the surgery. All patients performed the postoperative pulmonary rehabilitation, which consisted of positioning, stretching of respiratory muscles and thoracic cage, deep diaphragmatic breathing, coughing and huffing, and early mobilization from the first postoperative day. When possible, 6MWD, muscle strength, HADS, and CAT were re-evaluated 14 days after the operation.

### Statistical analysis

Data were expressed as means ± SD or median (range). The paired *t*-test or Fisher’s exact test was used to evaluate statistical significance. When data failed a normality test, the Mann-Whitney U test was performed. Correlations between variables were analyzed using Spearman’s rank correlation coefficient. Regression analysis was used for univariate analysis and multiple regression analysis (forced entry method) was used for multivariate analysis. All analyses were conducted using SPSS ver. 19 (SPSS Inc., Tokyo, Japan). *P* < 0.05 was considered statistically significant.

## Results

### Clinical characteristics

The characteristics and results of preoperative pulmonary function and laboratory tests of the 34 patients are shown in Table 1. The patients were predominantly male (76.4 %), had a high rate of smoking history (91.2 %), and squamous cell carcinoma (97.1 %). COPD was present in 12 patients (35.3 %). The preoperative clinical stage was 0-I in 12 patients, II in 4 patients, III in 16 patients, and IV in 2 patients. Twenty-two patients (64.7 %) received some sort of induction therapy (chemotherapy or chemoradiotherapy). Thirty-one patients (91.2 %) underwent open surgery. Three (8.8 %) patients experienced postoperative pulmonary complications. Two patients developed pneumonia (Clavien-Dindo Grade II) and one patient pneumothorax (Grade I). There was no in-hospital death or reoperation after esophagectomy.

**Table 1:** Clinical characteristics of 34 subjects

Variable	
Sex, male/female	*N* = 26/8
Age, years (range)	67.3 ± 8.1 (50–84)
Height, cm	163.2 ± 7.9
Weight, kg	54.9 ± 7.6
Body mass index	20.6 ± 2.2
Smoking history	*N* = 31
Squamous cell carcinoma/adenocarcinoma	*N* = 33/1
Clinical stage, 0-I/II/III/IV	*N* = 12/4/16/2
Lymphadenectomy, 1/2/3 fields	*N* = 1/24/9
Preoperative adjuvant therapy	*N* = 22
COPD	*N* = 12
%VC	101.5 ± 14.1
%FEV_1_	94.0 ± 15.9
FEV_1_/FVC, %	73.0 ± 7.9
Open surgery/video-assisted thoracic surgery	*N* = 31/3
Operation time, min	520 ± 90
Blood loss during surgery, ml	719 ± 456
Intensive Care Unit stay, days	1.3 ± 0.6
Timing of extubation, 0/1/2 postoperative days	*N* = 23/9/2
Hospital stay after surgery, days	21.2 ± 15.0
Walking with support, postoperative days	1.9 ± 1.8

Values are numbers (N) or mean ± SD. Total numbers of subjects are 34. *COPD* chronic obstructive pulmonary disease, *VC* vital capacity, *FEV*_*1*_ forced expiratory volume in one second, *FVC* forced vital capacity

### Correlations between preoperative parameters

Correlations between various parameters before the surgery are shown in Table 2. The preoperative 6MWD significantly correlated with hand grip strength (right: *r* = 0.562, *P* = 0.001, left: *r* = 0.511, *P* = 0.002), isometric knee extensor muscle strength (right: *r* = 0.482, *P* = 0.006, left: *r* = 0.535, *P* = 0.002), and SF-36 components, physical functioning (*r* = 0.454, *P* = 0.013), general health (*r* = 0.465, *P* = 0.011), mental health (*r* = 0.382, *P* = 0.045), and MCS (*r* = 0.497, *P* = 0.010). CAT scores showed significant inverse correlation with hand grip strength (right: *r* = -0.490, *P* = 0.005, left: *r* = -0.406, *P* = 0.002) and isometric knee extensor muscle strength (right: *r* = -0.491, *P* = 0.008, left: *r* = -0.565, *P* = 0.002). Correlations between QOL parameters or 6MWD and pulmonary functions before surgery are shown in Table 3. Correlations between SF-36 scores and CAT or HADS before surgery are shown in Table 4. Total CAT scores showed significant inverse correlation with most SF-36 components: physical functioning (*r* = -0.384, *P* = 0.040), general health (*r* = -0.432, *P* = 0.019), social functioning (*r* = -0.658, *P* < 0.001), vitality (*r* = -0.503, *P* = 0.005), role emotional (*r* = -0.381, *P* = 0.041), mental health (*r* = -0.510, *P* = 0.006), MCS (*r* = -0.584, *P* = 0.002), and RCS (*r* = -0.433, *P* = 0.027). HADS anxiety and depression significantly correlated with most SF-36 components. In contrast, HADS anxiety and depression did not significantly correlate with CAT scores.

**Table 2:** Correlation between preoperative parameters

Parameters		6MWD	Hand grip strength		Isometric knee extensor muscle strength	
			Right	Left	Right	Left
6MWD	r	1	0.562	0.511	0.482	0.535
	*P* value	0	0.001 (*n* = 34)	0.002 (*n* = 34)	0.006 (*n* = 31)	0.002 (*n* = 31)
CAT scores	r	−0.332	−0.490	−0.406	−0.491	−0.565
	*P* value	0.068 (*n* = 31)	0.005 (*n* = 31)	0.024 (*n* = 31)	0.008 (*n* = 28)	0.002 (*n* = 28)
SF-36 component						
Physical functioning	r	0.454	0.137	0.258	0.350	0.365
	*P* value	0.013 (*n* = 29)	0.478 (*n* = 29)	0.177 (*n* = 29)	0.080 (*n* = 26)	0.067 (*n* = 26)
Role physical	r	0.334	0.413	0.490	0.589	0.473
	*P* value	0.076 (*n* = 29)	0.026 (*n* = 29)	0.007 (*n* = 29)	0.002 (*n* = 26)	0.015 (*n* = 26)
Bodily pain	r	0.126	0.123	0.166	0.172	0.125
	*P* value	0.516 (*n* = 29)	0.525 (*n* = 29)	0.389 (*n* = 29)	0.401 (*n* = 26)	0.542 (*n* = 26)
General health	r	0.465	0.382	0.367	0.478	0.376
	*P* value	0.011 (*n* = 29)	0.041 (*n* = 29)	0.050 (*n* = 29)	0.014 (*n* = 26)	0.058 (*n* = 26)
Social functioning	r	0.334	0.221	0.221	0.370	0.306
	*P* value	0.077 (*n* = 29)	0.249 (*n* = 29)	0.248 (*n* = 29)	0.063 (*n* = 26)	0.128 (*n* = 26)
Vitality	r	0.287	0.356	0.398	0.433	0.283
	*P* value	0.131 (*n* = 29)	0.058 (*n* = 29)	0.034 (*n* = 29)	0.027 (*n* = 26)	0.161 (*n* = 26)
Role emotional	r	0.243	0.197	0.317	0.515	0.385
	*P* value	0.204 (*n* = 29)	0.305 (*n* = 29)	0.093 (*n* = 29)	0.007 (*n* = 26)	0.052 (*n* = 26)
Mental health	r	0.382	0.124	0.317	0.355	0.310
	*P* value	0.045 (*n* = 28)	0.529 (*n* = 28)	0.093 (*n* = 28)	0.081 (*n* = 25)	0.131 (*n* = 25)
PCS	r	0.159	0.345	0.346	0.284	0.228
	*P* value	0.436 (*n* = 26)	0.084 (*n* = 26)	0.083 (*n* = 26)	0.189 (*n* = 23)	0.296 (*n* = 23)
MCS	r	0.497	0.220	0.180	0.354	0.349
	*P* value	0.010 (*n* = 26)	0.281 (*n* = 26)	0.379 (*n* = 26)	0.097 (*n* = 23)	0.103 (*n* = 23)
RCS	r	0.292	0.225	0.345	0.587	0.435
	*P* value	0.148 (*n* = 26)	0.269 (*n* = 26)	0.084 (*n* = 26)	0.003 (*n* = 23)	0.038 (*n* = 23)

Spearman’s rank correlation coefficient was used for the analysis. *6MWD* 6-min walk distance, *CAT* COPD assessment test, *SF-36* the MOS 36-item Short-Form Health Survey Questionnaire, *PCS* physical component summary, *MCS* mental component summary, *RCS*, role/social component summary

**Table 3:** Correlation between preoperative parameters and pulmonary functions

Parameters		VC	%VC	FEV_1_	%FEV_1_	FEV_1_/FVC
6MWD (*n* = 34)	r	0.502	0.098	0.579	0.015	0.291
	*P* value	0.002	0.581	<0.001	0.934	0.095
CAT scores (*n* = 31)	r	− 0.418	− 0.334	− 0.415	− 0.269	− 0.265
	*P* value	0.019	0.066	0.020	0.143	0.149
SF-36 component						
Physical functioning (*n* = 29)	r	0.367	0.310	0.423	0.184	0.184
	*P* value	0.050	0.102	0.022	0.339	0.340
Role physical (*n* = 29)	r	0.403	0.009	0.197	− 0.315	− 0.236
	*P* value	0.030	0.964	0.305	0.096	0.218
Bodily pain (*n* = 29)	r	− 0.001	− 0.050	− 0.027	− 0.246	− 0.220
	*P* value	0.997	0.798	0.891	0.199	0.251
General health (*n* = 29)	r	0.174	− 0.079	0.142	− 0.223	− 0.070
	*P* value	0.367	0.684	0.462	0.244	0.717
Social functioning (*n* = 29)	r	0.177	0.275	0.210	0.180	0.106
	*P* value	0.358	0.149	0.275	0.350	0.585
Vitality (*n* = 29)	r	0.329	0.112	0.255	− 0.122	− 0.099
	*P* value	0.081	0.562	0.182	0.527	0.611
Role emotional (*n* = 29)	r	0.268	0.007	0.150	− 0.186	− 0.197
	*P* value	0.160	0.973	0.437	0.335	0.307
Mental health (*n* = 28)	r	0.334	0.310	0.368	0.245	0.212
	*P* value	0.082	0.108	0.054	0.208	0.279
PCS (*n* = 26)	r	0.095	− 0.136	0.022	− 0.426	− 0.276
	*P* value	0.645	0.508	0.916	0.030	0.173
MCS (*n* = 28)	r	0.217	0.284	0.291	0.200	0.157
	*P* value	0.286	0.159	0.149	0.327	0.443
RCS (*n* = 26)	r	0.435	0.075	0.290	− 0.165	− 0.121
	*P* value	0.026	0.717	0.150	0.420	0.556

Spearman’s rank correlation coefficient was used for the analysis. *VC* vital capacity, *%VC* % of the predicted value for VC, *FEV*_*1*_ forced expiratory volume in 1 s, *%FEV*_*1*_ % of the predicted value for FEV_1_, *FVC* forced vital capacity

**Table 4:** Correlation between SF-36 and CAT or HADS before surgery

SF-36 component		CAT	HADS anxiety	HADS depression
Physical functioning	r	−0.384	−0.339	−0.500
	*P* value	0.040	0.072	0.006
Role physical	r	−0.323	−0.745	−0.557
	*P* value	0.088	<0.001	0.002
Bodily pain	r	−0.167	−0.368	−0.252
	*P* value	0.385	0.049	0.187
General health	r	−0.432	−0.543	−0.321
	*P* value	0.019	0.002	0.090
Social functioning	r	−0.658	−0.464	−0.403
	*P* value	<0.001	0.011	0.030
Vitality	r	−0.503	−0.448	−0.554
	*P* value	0.005	0.015	0.002
Role emotional	r	−0.381	−0.593	−0.470
	*P* value	0.041	0.001	0.010
Mental health (*n* = 28)	r	−0.510	−0.379	−0.563
	*P* value	0.006	0.047	0.002
PCS (*n* = 26)	r	−0.245	−0.364	−0.266
	*P* value	0.228	0.067	0.188
MCS (*n* = 26)	r	−0.584	−0.361	−0.317
	*P* value	0.002	0.070	0.115
RCS (*n* = 26)	r	−0.433	−0.653	−0.648
	*P* value	0.027	<0.001	<0.001

Spearman’s rank correlation coefficient was used for the analysis. *N* = 29, otherwise indicated. *HADS* the Hospital Anxiety and Depression Scale

### Correlations between postoperative parameters

Correlations between various parameters after the surgery are shown in Table 5. The postoperative 6MWD significantly correlated with postoperative left isometric knee muscle extensor strength (*r* = 0.526, *P* = 0.003).

**Table 5:** Correlation between postoperative parameters

Parameters		6MWD	Hand grip strength		Isometric knee extensor muscle strength	
			Right	Left	Right	Left
6MWD	r	1	0.159	0.071	0.325	0.526
	*P* value	0	0.394 (*n* = 31)	0.704 (*n* = 31)	0.085 (*n* = 29)	0.003* (*n* = 29)
CAT scores	r	−0.053	−0.020	0.014	−0.421	−0.383
	*P* value	0.816 (*n* = 22)	0.927 (*n* = 23)	0.948 (*n* = 23)	0.058 (*n* = 21)	0.086 (*n* = 21)

Spearman’s rank correlation coefficient was used for the analysis. *P < 0.05

### Comparison between pre- and postoperative measurements

Next, the effects of esophagectomy on physical and psychological status were examined. Comparisons and changes of 6MWD, desaturation during the 6MWT, muscle strength, HADS, and CAT before and after surgery are shown in Table 6. The post-operative 6MWD (409 ± 108 m) was significantly shorter than the pre-operative value (496 ± 76 m, *P* < 0.001). Muscle strength results showed that both right and left hand grip strengths were significantly decreased after the operation (*P* = 0.01). Left side isometric knee extensor muscle strength was significantly decreased after the operation (*P* = 0.02). That of the right side was also decreased after the operation, but the difference was not statistically significant level (*P* = 0.08). The number of patients whose HADS anxiety score was ≥8 increased from 4 to 8 after surgery, although the difference did not reach a statistically significant level by Fisher’s exact test (*P* = 0.31). CAT scores significantly increased after surgery (*P* = 0.02).

**Table 6:** Comparison of 6MWD, muscle strength, and HADS before and after surgery

	Before	After surgery	*P* value	Changes
6MWD, m (*n* = 32)	494 ± 76	409 ± 108	0.001 > *	−85 ± 88
Desaturation during 6MWT^a^	*N* = 5	*N* = 5	1	
Hand grip strength, kgf (*n* = 33)				
Right	30.6 ± 9.1	28.7 ± 8.6	0.01*	−1.9 ± 3.9
Left	28.8 ± 8.6	27.5 ± 7.7	0.01*	−1.3 ± 2.8
Isometric knee extensor muscle strength, kgf (*n* = 31)				
Right	26.0 ± 8.5	24.7 ± 8.2	0.08	−1.3 ± 3.8
Left	25.2 ± 9.4	22.9 ± 7.8	0.02*	−2.3 ± 4.9
HADS anxiety (*n* = 23)	5.4 ± 2.9	5.1 ± 4.0	0.76	−0.3 ± 4.0
Scores ≥ 8	*N* = 4	*N* = 8	0.31	
HADS depression (*n* = 23)	5.7 ± 3.7	5.4 ± 3.8	0.54	−0.3 ± 2.3
Scores ≥ 8	*N* = 6	*N* = 7	1	
CAT (*n* = 24)	10.4 ± 5.6	15.9 ± 7.3	0.02*	5.5 ± 7.5

Values are mean ± SD and compared by paired t-test or Fisher’s exact test. ^a^Desaturation was defined as a fall in SpO_2_ ≥ 4 % or SpO_2_ < 90 % during the 6MWT. *P <0.05

Two different surgical techniques, open surgery for 31 patients and video-assisted thoracic (closed) surgery for three patients, were performed (Table 1). Demographics and data of pre- and postoperative measurements of three patients who underwent video-assisted thoracic surgery are shown in Additional file 1: Table S1. The mean values of postoperative 6MWD and changes of 6MWD after the surgery were 364 m and −60 m. The mean changes of right and left hand grip strengths and right isometric knee extensor muscle strength were −3.8 kgf, −2.5 kgf, and −2.3 kgf, respectively. Left isometric knee extensor muscle strength was increased in two patients, and the mean changes were 1.0 kgf.

### Effects of COPD and preoperative chemoradiotherapy

The effects of comorbidity of COPD on physical and QOL parameters were examined. The parameters of patients with and without COPD are compared in Table 7. Preoperative %FEV_1_ (*P* < 0.001), FEV_1_/FVC (*P* < 0.001), postoperative 6MWD (*P* = 0.034), and preoperative left isometric knee extensor muscle strength (*P* = 0.018) were significantly lower in patients with COPD than those without COPD. There was no significant difference in preoperative SF-36 components between the groups.

**Table 7:** Comparison of data with and without COPD

	Non-COPD (*n* = 22)	COPD (*n* = 12)	*P* value
Pre %FEV_1_	101.3 ± 12.7	75.2 ± 21.1	<0.001*
Pre FEV_1_/FVC, %	77.3 ± 5.7	60.5 ± 16.3	<0.001*
Pre 6MWD, m	509 ± 80	466 ± 60	0.127
Desaturation during 6MWT^a^	*N* = 3	*N* = 2	0.580
Post 6MWD, m	438 ± 100 (*n* = 21)	351 ± 105 (*n* = 11)	0.034*
Desaturation during 6MWT^a^	*N* = 2	*N* = 3	0.290
Changes in 6MWD, m	−70 ± 83 (*n* = 21)	−114 ± 98 (*n* = 11)	0.123
Pre hand grip strength, kgf			
Right	32.4 ± 8.4	28.4 ± 10.5	0.204
Left	30.7 ± 8.8	27.0 ± 9.4	0.204
Post hand grip strength, kgf			
Right	29.6 ± 7.9 (*n* = 21)	26.6 ± 9.7	0.152
Left	28.7 ± 7.3 (*n* = 21)	25.8 ± 8.0	0.242
Pre isometric knee extensor muscle strength, kgf			
Right	26.7 ± 8.2 (*n* = 20)	24.7 ± 9.4 (*n* = 11)	0.502
Left	27.6 ± 8.9 (*n* = 20)	20.1 ± 9.1 (*n* = 11)	0.018*
Post isometric knee extensor muscle strength, kgf			
Right	25.8 ± 8.3 (*n* = 20)	22.8 ± 8.0 (*n* = 11)	0.183
Left	24.9 ± 7.7 (*n* = 20)	19.3 ± 6.9 (*n* = 11)	0.020*
Pre HADS anxiety	6.1 ± 2.8 (*n* = 21)	4.5 ± 3.2 (*n* = 10)	0.135
Scores ≥ 8	*N* = 5	*N* = 2	1
Pre HADS depression	5.6 ± 3.2 (*n* = 21)	5.5 ± 4.1 (*n* = 10)	0.852
Scores ≥ 8	*N* = 5	*N* = 4	0.781
Post HADS anxiety	4.8 ± 3.3 (*n* = 15)	5.6 ± 5.4 (*n* = 8)	0.925
Scores ≥ 8	*N* = 4	*N* = 4	0.676
Post HADS depression	5.7 ± 3.8 (*n* = 15)	4.9 ± 4.1 (*n* = 8)	0.681
Scores ≥ 8	*N* = 5	*N* = 2	1
Pre CAT (*n* = 31)	8.1 ± 5.5 (*n* = 21)	14.2 ± 5.5 (*n* = 10)	0.008*
Post CAT (*n* = 24)	14.1 ± 5.3 (*n* = 16)	19.6 ± 9.6 (*n* = 8)	0.136
Pre SF-36 component			
Physical functioning	49.5 ± 15.1 (*n* = 19)	40.9 ± 15.5 (*n* = 10)	0.308
Role physical	37.6 ± 19.3 (*n* = 19)	38.1 ± 21.6 (*n* = 10)	0.456
Bodily pain	44.8 ± 12.9 (*n* = 19)	41.4 ± 24.5 (*n* = 10)	0.804
General health	45.3 ± 9.5 (*n* = 19)	45.4 ± 11.4 (*n* = 10)	0.946
Vitality	43.2 ± 17.6 (*n* = 19)	39.0 ± 18.4 (*n* = 10)	0.804
Social functioning	49.0 ± 11.2 (*n* = 19)	38.8 ± 17.3 (*n* = 10)	0.069
Role emotional	41.3 ± 18.2 (*n* = 19)	40.7 ± 17.0 (*n* = 10)	0.604
Mental health	49.3 ± 14.3 (*n* = 19)	38.4 ± 12.7 (*n* = 9)	0.061
PCS	46.7 ± 5.8 (*n* = 19)	52.5 ± 9.7 (*n* = 7)	0.209
MCS	51.2 ± 10.1 (*n* = 19)	45.5 ± 9.6 (*n* = 7)	0.073
RCS	38.1 ± 12.4 (*n* = 19)	36.3 ± 23.3 (*n* = 7)	0.955

Values are mean ± SD and compared by paired t-test, Mann-Whitney U test or Fisher’s exact test. *P < 0.05. ^a^Desaturation was defined as a fall in SpO_2_ ≥ 4 % or SpO_2_ < 90 %. The HADS score ≥8 denotes a probable case. *Pre* preoperative, *Post* postoperative

We also compared the results of physical and QOL parameters of patients with and without preoperative chemotherapy or radiotherapy (Table 8). Among preoperative SF-36 components, role emotional and PCS scores of patients undergoing chemoradiotherapy were significantly lower than those without (*P* = 0.019). There was no significant difference in other parameters between the groups.

**Table 8:** Comparison of data with and without chemoradiotherapy

	Without (*n* = 12)	Chemotherapy and/or radiotherapy (*n* = 22)	*P* value
%FEV_1_	93.3 ± 20.4	94.5 ± 13.9	0.466
FEV_1_/FVC, %	69.8 ± 7.7	74.8 ± 7.8	0.136
Pre 6MWD, m	485 ± 59	499 ± 82	0.790
Desaturation during 6MWT^a^	*N* = 4	*N* = 1	1
Post 6MWD, m	389 ± 109	421 ± 109 (*n* = 20)	0.654
Desaturation during 6MWT^a^	*N* = 0	*N* = 5	0.155
Changes in 6MWD, m	−97 ± 61	−78 ± 104 (*n* = 20)	0.158
Pre hand grip strength, kgf			
Right	33.0 ± 12.0	29.9 ± 7.4	0.327
Left	31.8 ± 11.0	28.1 ± 7.8	0.261
Post hand grip strength, kgf			
Right	30.8 ± 11.3 (*n* = 11)	27.7 ± 7.0	0.560
Left	28.4 ± 8.9 (*n* = 11)	27.1 ± 7.2	0.693
Pre isometric knee extensor muscle strength, kgf			
Right	30.0 ± 9.4	23.4 ± 7.0 (*n* = 20)	0.059
Left	28.8 ± 11.6	23.0 ± 7.2 (*n* = 20)	0.269
Post isometric knee extensor muscle strength, kgf			
Right	28.3 ± 9.0	22.5 ± 7.0 (*n* = 20)	0.120
Left	25.4 ± 8.8	21.3 ± 6.8 (*n* = 20)	0.287
Pre HADS anxiety	4.9 ± 3.5	6.0 ± 2.6 (*n* = 19)	0.367
Scores ≥8	*N* = 3	*N* = 4	1
Pre HADS depression	4.5 ± 2.9	6.2 ± 3.7 (*n* = 19)	0.287
Scores ≥8	*N* = 3	*N* = 5	1
Post HADS anxiety	5.3 ± 4.8 (*n* = 7)	5.0 ± 3.8 (*n* = 16)	1
Scores ≥8	*N* = 2	*N* = 6	1
Post HADS depression	3.3 ± 2.6 (*n* = 7)	6.3 ± 4.0 (*n* = 16)	0.103
Scores ≥8	*N* = 2	*N* = 9	0.682
Pre CAT (*n* = 31)	9.3 ± 6.4	10.6 ± 6.1 (*n* = 19)	0.509
Post CAT (*n* = 24)	13.7 ± 8.6 (*n* = 6)	16.7 ± 6.9 (*n* = 18)	0.343
Pre SF-36 component			
Physical functioning	51.7 ± 20.1 (*n* = 11)	43.4 ± 11.6 (*n* = 18)	0.146
Role physical	46.2 ± 25.3 (*n* = 11)	32.6 ± 13.8 (*n* = 18)	0.084
Bodily pain	38.7 ± 21.0 (*n* = 11)	46.7 ± 14.6 (*n* = 18)	0.387
General health	49.2 ± 7.9 (*n* = 11)	42.9 ± 10.6 (*n* = 18)	0.055
Vitality	48.1 ± 19.0 (*n* = 11)	37.9 ± 16.1 (*n* = 18)	0.188
Social functioning	45.0 ± 18.1 (*n* = 11)	45.7 ± 11.7 (*n* = 18)	0.580
Role emotional	50.2 ± 19.7 (*n* = 11)	35.5 ± 13.7 (*n* = 18)	0.049*
Mental health	47.1 ± 17.4 (*n* = 10)	45.1 ± 13.2 (*n* = 18)	1.000
PCS	53.2 ± 6.6 (*n* = 8)	46.0 ± 6.6 (*n* = 18)	0.019*
MCS	48.9 ± 8.5 (*n* = 8)	50.0 ± 10.8 (*n* = 18)	0.807
RCS	44.3 ± 11.3 (*n* = 8)	32.5 ± 16.0 (*n* = 18)	0.090

Values are mean ± SD and compared by paired t-test, Mann-Whitney U test or Fisher’s exact test. ^a^Desaturation was defined as a fall in SpO_2_ ≥ 4 % or SpO_2_ < 90 %

### Factors affecting postoperative 6MWD

Next, we aimed to identify pre- and intraoperative factors affecting the postoperative loss of exercise capacity. Changes in 6MWD before and after surgery were chosen because the 6MWT is a validated method to assess functional exercise capacity and efficacy of pulmonary rehabilitation [27]. Results of the univariate regression analysis showed that the preoperative PCS, a component of SF-36, significantly correlated with decrease of 6MWD (*r* = 0.437, *P* = 0.033) (Table 9). On the other hand, preoperative 6MWD, pulmonary functions, muscle strength, COPD comorbidity, chemoradiotherapy, blood loss during surgery, and operation time did not predict changes in 6MWD (Table 9). Multiple regression analysis including all relevant variables with *P* values <0.10 in univariate analysis showed that only PCS significantly correlated with changes in 6MWD (*r* = 0.488, *P* = 0.027) (Table 9). There was a trend toward a greater risk of loss of 6MWD as preoperative %VC decreased (*P* = 0.077).

**Table 9:** Correlations between changes in 6MWD after esophagectomy and other parameters

Parameters	Univariate analysis		Multivariate analysis	
	Standardized partial regression coefficient	*P* value	Standardized partial regression coefficient	*P* value
Preoperative parameters				
Body mass index	−0.056	0.760	ND	ND
%VC	−0.327	0.068	−0.573 (*n* = 23)	0.077
%FEV_1_	−0.302	0.093	0.369 (*n* = 23)	0.273
6MWD	0.153	0.403	ND	ND
Hand grip strength				
Right	0.273	0.130	ND	ND
Left	0.254	0.162	ND	ND
Isometric knee extensor muscle strength				
Right (*n* = 29)	0.028	0.885	ND	ND
Left (*n* = 29)	−0.101	0.601	ND	ND
CAT (*n* = 29)	0.220	0.251	ND	ND
SF-36 component				
Physical functioning (*n* = 27)	−0.186	0.353	ND	ND
Role physical (*n* = 27)	−0.008	0.968	ND	ND
Bodily pain (*n* = 27)	0.283	0.153	ND	ND
General health (*n* = 27)	0.049	0.809	ND	ND
Social functioning (*n* = 27)	−0.217	0.276	ND	ND
Vitality (*n* = 27)	0.210	0.293	ND	ND
Role emotional (*n* = 27)	0.028	0.891	ND	ND
Mental health (*n* = 26)	−0.145	0.480	ND	ND
PCS (*n* = 24)	0.437	0.033*	0.488 (*n* = 23)	0.027*
MCS (*n* = 24)	−0.073	0.734	ND	ND
RCS (*n* = 24)	0.115	0.593	ND	ND
With-without COPD	0.241	0.184	ND	ND
With-without chemo-radio therapy	−0.103	0.575	ND	ND
Intraoperative parameters				
Blood loss during surgery	0.145	0.428	ND	ND
Operation time	0.145	0.430	ND	ND
Postoperative parameters				
Postoperative hospital stays	0.046	0.802	ND	ND
Changes in hand grip strength				
Right (*n* = 31)	0.153	0.412	ND	ND
Left (*n* = 31)	0.143	0.442	ND	ND
Changes in isometric knee extensor muscle strength				
Right (*n* = 29)	0.045	0.815	ND	ND
Left (*n* = 29)	0.089	0.646	ND	ND
Changes in CAT scores (*n* = 23)	−0.219	0.315	ND	ND

The multivariate analysis includes relevant variables with *P* values <0.10 in univariate analysis (preoperative %VC and %FEV_1_, and PCS). *ND* not done. *P < 0.05. *N* = 32 otherwise indicated

## Discussion

The main findings of the present study of patients with esophageal cancer who underwent esophagectomy with perioperative pulmonary rehabilitation were: 1) 6MWD and skeletal muscle strength had significantly decreased and CAT scores had increased 2 weeks after surgery, 2) PCS, a component of SF-36, significantly correlated with the decrease of 6MWD, and 3) comorbidity of COPD had a significant impact on health-related QOL, muscle strength, and 6MWD. To our knowledge, this is the first study to characterize the status of physical fitness, exercise capacity, health-related QOL, anxiety, and depression in patients with esophageal cancer before and after the surgery.

The 6MWT is a simple but well validated method to assess functional exercise capacity in patients with pulmonary and cardiovascular diseases [22] and the efficacy of pulmonary rehabilitation [27]. In the present study, preoperative 6MWD positively correlated with hand grip and isometric knee extensor muscle strength. It is expected that postoperative pulmonary rehabilitation helped maintain and recover the physical status of patients. However, the postoperative 6MWD and muscle strength were significantly lower than the preoperative values. Importantly, a mean decrease in the 6MWD of 85 m exceeded the clinically important distance of 54 m [28], demonstrating the significant short-term impact of esophagectomy on exercise capacity. Even in the three patients who underwent video-assisted thoracic surgery, the mean decrease in 6MWD was 60 m, above the clinically important value. Our results were consistent with the previous report by Tatematsu et al. that 6MWD and knee-extensor muscle strength, which were measured on the day of hospital discharge (median 21 days after surgery), were significantly decreased after esophagectomy [13]. Tatematsu et al. also reported that the change in the physical functional score on EORTC QLQ-C30 was significantly affected by that in the 6MWD by multiple regression analysis [13]. In our study, results of multiple and univariate regression analysis show that the loss of 6MWD was significantly affected by the preoperative SF-36 physical component PCS. In contrast, comorbidity of COPD, preoperative adjuvant therapy, pulmonary function test results, operation duration, or blood loss during surgery did not affect the change in 6MWD, consistent with the results of Tatematsu et al. These findings indicate that the 6MWT is a convenient and useful tool to assess the physical status in order to plan a perioperative rehabilitation program for patients with esophageal cancer.

Long-term cigarette smoking is an important risk factor for developing COPD as well as squamous cell carcinoma of the esophagus. It has been reported that COPD is one of the predictors of postoperative mortality after esophagectomy [5]. In our study, 91.2 % of patients were smokers, and the prevalence of COPD was relatively high (35.3 %). Moreover, both physical and QOL parameters were affected by comorbidity with COPD. The CAT is a validated questionnaire designed to assess and quantify the impact of COPD symptoms on health-related QOL [26]. The CAT scores strongly correlate with scores on the St. George Respiratory Questionnaire, a tool to evaluate the respiratory-related QOL [26]. Significant correlations between scores of total CAT and SF-36, a measure of the general health-related QOL, were found in our cohort, consistent with findings in COPD patients [29]. Preoperative CAT scores were significantly higher and postoperative 6MWD and left hand grip strength were significantly lower in patients with COPD than those without. In addition, postoperative CAT scores were significantly higher than preoperative scores, suggesting the impact of esophagectomy on respiratory-related QOL. Although the use of CAT for assessment of the respiratory-related QOL after surgery such as esophagectomy has not been validated yet, CAT was used before and after the surgery for the following reasons. First, almost all the patients in our cohort were smokers with a high prevalence of COPD. Second, pulmonary rehabilitation was the main objective of our perioperative rehabilitation program in order to prevent pulmonary complications after esophagectomy. Third, a recent study demonstrated that the CAT is beneficial to assess respiratory symptom and complications even in smokers without COPD [30]. Future studies are required to validate the use of CAT to assess respiratory-related QOL in patients who receive perioperative pulmonary rehabilitation. It would also be useful to evaluate the correlation between scores of CAT and QOL questionnaires specific for cancer and esophageal diseases such as the EORTC QLQ-C30 [14] and EORTC QLQ-OES24 [15].

The long-term impacts of esophagectomy on physical, QOL, or mental status after discharge have been investigated by other groups [11, 15, 31, 32]. In contrast, there is little information on postoperative physical and QOL status before discharge. The major purpose of our study was to evaluate how the physical, mental, and QOL parameters have recovered 2 weeks after esophagectomy during the hospital stay. Severer cases were excluded from the analysis because of gait disturbance 2 weeks after the surgery (Fig. 1). The incidence of pulmonary complications (8.8 %) was lower than that in previous reports (15–36 %) [4, 33, 34]. Thus, our patients reflect a relatively good clinical course shortly after the esophagectomy.

This study has several limitations. The data were collected retrospectively from patients who underwent esophagectomy. We did not aim to determine the factors that predict postoperative complications or examine the effectiveness of perioperative pulmonary rehabilitation either. It is impossible to clarify whether the rehabilitation was beneficial for health outcomes after the esophagectomy from the present study design. A previous study demonstrated that intensive prehabilitation reduced postoperative pulmonary complications after esophagectomy [10], indicating that improvement of physical fitness before the surgery may lead to better clinical outcomes after esophagectomy. Our findings demonstrated both the physical and QOL status of in-hospital patients two weeks after esophagectomy with perioperative rehabilitation. Further improvements, both from the viewpoint of minimally invasive surgery and perioperative management, are warranted. Prospective studies with a larger number of subjects including patients both with and without pulmonary rehabilitation are necessary to characterize the risk factors of postoperative complications and to improve the perioperative rehabilitation programs.

## Conclusion

Our results indicate that esophagectomy is detrimental to health-related QOL and physical fitness at two weeks after surgery. Further investigation is required to establish a strategy for perioperative rehabilitation to improve postoperative outcomes.

## Additional files

Additional file 1:
**Table S1.** Data of three patients who underwent video-assisted thoracic surgery. (DOCX 19 kb)
Additional file 2:
**Table S2.** The datasets of 34 patients. (XLSX 23 kb)

## Abbreviations

6MWD: 6-min walk distance
6MWT: 6-min walk test
CAT: COPD assessment test
COPD: Chronic obstructive pulmonary disease
EORTC QLQ: European Organization for the Research and Treatment of Cancer QOL Core Questionnaire
FEV_1_: Forced expiratory volume in one second
FVC: Forced vital capacity
HADS: Hospital Anxiety and Depression Scale
MCS: Mental component summary
PCS: Physical component summary
QOL: Quality of life
RCS: Role/social component summary
SF-36: The MOS 36-item Short-Form Health Survey version 2
SpO_2_: Oxygen saturation of a peripheral artery
VC: Vital capacity
