# Supplementary material for: Changes in exercise capacity, muscle strength, and health-related quality of life in esophageal cancer patients undergoing esophagectomy
Source: BMC Sports Sci Med Rehabil. 2016 Nov 3;8:34. doi: 10.1186/s13102-016-0060-y (PMC5093971; doi:10.1186/s13102-016-0060-y)
Supplement: Additional file 1: — Table S1. Data of three patients who underwent video-assisted thoracic surgery. (DOCX 19 kb) [file 13102_2016_60_MOESM1_ESM.docx]

**Additional file 1: Table S1.**

|  | Case no. 3 | Case no. 8 | Case no. 11 | Mean |
| --- | --- | --- | --- | --- |
| Sex | Male | Female | Male |  |
| %FEV_1_ | 90.6 | 70.2 | 98.6 | 86.6 |
| FEV_1_/FVC, % | 77.7 | 65.6 | 71.8 | 71.7 |
| 6MWD, pre/post, m | 402/282 | 450/420 | 420/391 | 424/364 |
| Desaturation during 6MWT†, pre/post | Yes/No | No/No | Yes/No |  |
| Changes in 6MWD, m | -120 | -30 | -29 | -60 |
| Hand grip strength, pre/post, kgf |  |  |  |  |
| Right | 38.1/28.5 | 23.1/23.8 | 31.1/28.8 | 30.8/27.0 |
| Left | 35.5/31.0 | 23.5/23.4 | 26.7/24.0 | 28.6/26.1 |
| Isometric knee extensor muscle strength, pre/post, kgf |  |  |  |  |
| Right | 26.4/25.1 | 21.9/20.2 | 31.1/27.4 | 26.5/24.2 |
| Left | 26.4/26.7 | 19.8/19.6 | 26.7/29.5 | 24.3/25.3 |
| HADS anxiety, pre/post | 11/ND | 5/3 | 7/8 | 7.7/- |
| HADS depression, pre/post | 3/ND | 7/6 | 10/11 | 6.7/- |
| CAT, pre/post | 12/ND | 12/ND | 7/6 | 10.3/- |

†Desaturation was defined as a fall in SpO_2_ ≥ 4% or SpO_2_ < 90%. ND, not done.
